# Precision-mapping and statistical validation of quantitative trait loci by machine learning

**DOI:** 10.1186/1471-2156-9-35

**Published:** 2008-05-02

**Authors:** Justin Bedo, Peter Wenzl, Adam Kowalczyk, Andrzej Kilian

**Affiliations:** 1 Life Sciences, NICTA and Department of Electrical and Electronic Engineering, The University of Melbourne, Parkville, Victoria 3010, Australia; 2 Diversity Arrays P/L, 1 Wilf Crane Cr. (Yarralumla), Canberra, ACT 2600, Australia; 3 The Research School of Information Sciences and Engineering, The Australian National University, Canberra, Australia

## Abstract

**Background:**

We introduce a QTL-mapping algorithm based on Statistical Machine Learning (SML) that is conceptually quite different to existing methods as there is a strong focus on generalisation ability. Our approach combines ridge regression, recursive feature elimination, and estimation of generalisation performance and marker effects using bootstrap resampling. Model performance and marker effects are determined using independent testing samples (individuals), thus providing better estimates. We compare the performance of SML against Composite Interval Mapping (CIM), Bayesian Interval Mapping (BIM) and single Marker Regression (MR) on synthetic datasets and a multi-trait and multi-environment dataset of the progeny for a cross between two barley cultivars.

**Results:**

In an analysis of the synthetic datasets, SML accurately predicted the number of QTL underlying a trait while BIM tended to underestimate the number of QTL. The QTL identified by SML for the barley dataset broadly coincided with known QTL locations. SML reported approximately half of the QTL reported by either CIM or MR, not unexpected given that neither CIM nor MR incorporates independent testing. The latter makes these two methods susceptible to producing overly optimistic estimates of QTL effects, as we demonstrate for MR. The QTL resolution (peak definition) afforded by SML was consistently superior to MR, CIM and BIM, with QTL detection power similar to BIM. The precision of SML was underscored by repeatedly identifying, at ≤ 1-cM precision, three QTL for four partially related traits (heading date, plant height, lodging and yield). The set of QTL obtained using a 'raw' and a 'curated' version of the same genotypic dataset were more similar to each other for SML than for CIM or MR.

**Conclusion:**

The SML algorithm produces better estimates of QTL effects because it eliminates the optimistic bias in the predictive performance of other QTL methods. It produces narrower peaks than other methods (except BIM) and hence identifies QTL with greater precision. It is more robust to genotyping and linkage mapping errors, and identifies markers linked to QTL in the absence of a genetic map.

## Background

The notion that DNA polymorphism explains the phenotypic diversity of living organisms has been the driving force behind the Human Genome Project and widespread investment in plant and animal genomics. Over the last 30 years, many examples of causal effects on phenotypes arising from DNA sequence variation have been reported. Finding associations between DNA variation and phenotypes is straightforward for 'simple' traits that are inherited in a Mendelian fashion as monogenic characters. Yet, most of the economically important phenotypic variation (e.g. crop yield and its components) is inherited through a number of Quantitative Trait Loci (QTL) with different magnitudes of effect and complex interactions among themselves and with the environment [1].

QTL can be identified through their genetic linkage with molecular markers. In a typical experiment, the progeny of an experimental population are simultaneously analysed for their genetic makeup (molecular markers) and one or more phenotypic traits of interest. The marker data are used to build a genetic map, which is a pre-requisite for the majority of QTL-detection methods [2,3]. The simplest method to identify markers linked to QTL is single Marker Regression (MR), which fits a linear model to each marker using the trait data. Simple Interval Mapping (SIM) disentangles QTL effects from the confounding effect of linkage distance between markers and QTL by regressing phenotypic data on the genotypic information for marker intervals rather than the markers themselves [4]. QTL are detected by 'stepping' through the whole genome to generate a profile of the proportion of phenotypic variance explained or the logarithm-of-odds ratio (LOD score) in favour of a QTL.

The Composite Interval Mapping (CIM) approach refines the SIM algorithm by incorporating background markers as cofactors into a multiple regression model [5]. In this way, variation due to other QTL can be partly accounted for. The CIM approach was further extended by using multiple marker intervals to fit multi-QTL models to the trait data and selecting the 'best' model with a stepwise forward and backward selection procedure (Multiple Interval Mapping; MIM) [6]. Other approaches such as Bayesian Interval Mapping (BIM) [7] approach the problem by applying Bayesian inference over the whole genome using priors designed to produce sparse models.

**Figure 1:**
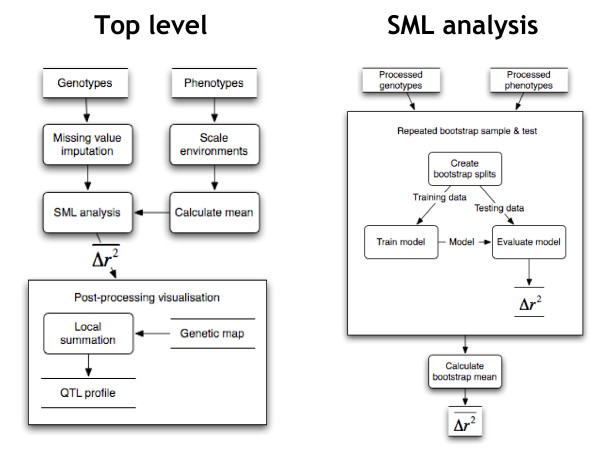
**System dataflow diagram**. Dataflow diagram (DFD) depicting the QTL analysis. Rectangles with round corners indicate processes, other rectangles indicate data stores, and lines indicate data flow. The left figure shows the top-level DFD, the right shows further detail of the 'SML analysis' process.

Here we explore a conceptually quite different QTL-mapping approach that focuses on generalisation ability. The approach is based on Statistical Machine Learning (SML) and differs from other methods in that it estimates the generalisation performance of a QTL model by splitting the data into independent training and testing subsets that are used for model induction and evaluation, respectively (Figure 1). Resampling data into training and testing subsets is quite common in microarray analyses, particularly in the context of cancer genomics [8,9].

Our QTL detection method determines the contribution of each marker to the model performance during the recursive feature elimination (RFE) procedure. First, a linear model containing every marker is fitted to the phenotype. The model is then reduced in size by recursively eliminating the least useful markers and refitting the model until only a single marker is left, which is similar to recursive feature elimination support vector machines [10,11]. We assign the *change in variance explained *after each elimination (measured on the test set) to the marker that was removed. The entire process is then repeated numerous times to derive an unbiased bootstrap estimate of the predictive power of each marker. To generate a QTL profile across the genome, the contributions of genetically linked markers within a sliding map window are added.

We compare the performance of the SML algorithm with the performance of two conventional QTL-mapping methods (MR, CIM) and the more recently developed BIM. For this purpose, we re-analyse a well-known multi-trait and multi-environment dataset for a population of doubled haploid (DH) lines derived from the F_1 _of a cross between cultivars Steptoe and Morex, and study some synthetic datasets.

## Results and Discussion

### Treatment of multi-environment data

In QTL mapping, we are primarily interested in quantifying the influence of genotypic variation on phenotypes. In practice, this is confounded by environmental variation to differing extents depending on the trait. In this paper, we limit our approach to mapping the genotypic component of the traits. The interaction between QTL and environments (QTL × E), an important element influencing phenotypic variation of many quantitative characters, will be addressed in a separate paper.

**Figure 2:**
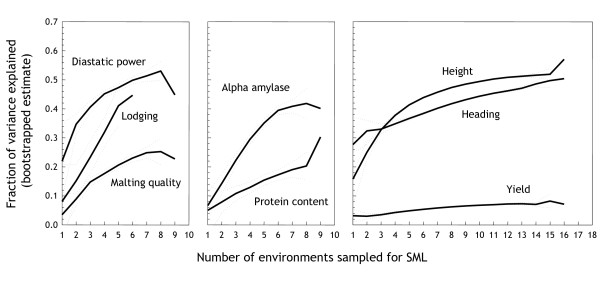
**Multiple environments**. Effect of including phenotypic data from multiple environments before modelling. Along the *x*-axis is the number of environments used in the pre-processing of phenotypic data, and the *y*-axis is the fraction of variance explained. For each number of environments, all possible permutations of the available environments were tested. Each permutation was evaluated by a 50-permutation bootstrap of a whole-genome model fitted using ridge regression. Dotted lines are 95% confidence intervals for the mean derived using the t-test.

In order to precisely measure the genotypic component we use data collected on genetically identical Steptoe/Morex DH lines grown in multiple environments. We standardise the phenotypes within each environment to a mean of 0 and a standard deviation of 1, and then calculate the mean (per phenotype and genotype) across all environments. The scaling within environments aligns the distributions, and the averaging provides an estimate of the common underlying signal. The resulting increase in QTL detection power for a whole-genome SML model based on 548 markers is demonstrated in Figure 2; incorporating information from multiple environments provides an increase in the variance explained for all traits.

The benefit from increasing the number of environments differs between traits. This is not surprising as more environments will provide a better estimate of the genotypic variation, thus traits that are heavily influenced by the environment are expected to benefit more from the inclusion of more environments. The latter is seen clearly for lodging, *α*-amylase, and plant height where the inclusion of more environments produces a substantial increase in performance over a single environment. We can therefore use the degree of increase in variance explained as a crude measure of environmental "susceptibility" or, conversely, heritability of the trait. For example, heading time appeared to be less influenced by environmental factors (2-fold increase in variance explained) than plant height (3.5-fold increase) and the degree of lodging (5.5-fold increase). The performance improvement due to the inclusion of multiple environments is, of course, accompanied by a decrease in the fraction of the total (multi-environment) variance that remains after averaging the scaled phenotypes across environments (Table 1), and thus the latter can also be used as an estimate of environmental susceptibility.

**Table 1:** Percentage of total phenotypic variance remaining after averaging scaled phenotypes across environments.^a^

**Trait**	**Variance (%)**
*α*-Amylase	52.2
Diastatic power	74.1
Malt extract	54.0
Heading date	70.4
Plant height	64.2
Lodging	40.3
Grain protein content	45.6
Yield	22.4

^a ^The number of environments was 6 for lodging; 9 for *α*-amylase, diastatic power, malt extract, and grain protein; and 16 for the remaining traits.

### Model size and genetic complexity of traits

The SML algorithm combines Recursive Feature (marker) Elimination (RFE) with ridge regression and bootstrapping (see *Methods*). It starts with a whole-genome model and progressively eliminates individual markers from the model. When the algorithm starts removing markers with predictive value, the predictive variance explained starts dropping. The number of markers in the smallest model that explains a close-to-maximum fraction of the variance (the 'optimal model') can therefore be used as an indicator of the genetic complexity of a trait.

**Figure 3:**
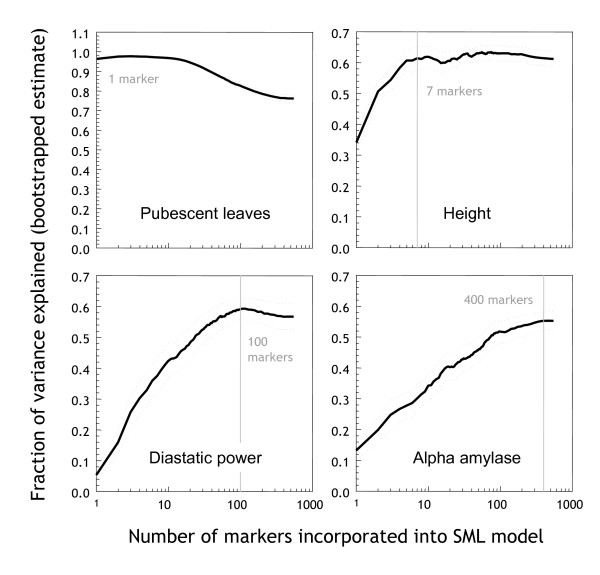
**Reduction of model size**. Performance of models of varying size (number of markers) for four traits: pubescent leaves, plant height, diastatic power, and *α*-amylase. The *x*-axis is the number of features (markers), and the *y*-axis is the fraction of variance explained, estimated using the zero bootstrap. Vertical grey lines indicate the optimal operating points. Dotted lines are 95% confidence intervals derived using the t-test.

Figure 3 displays the performance of models of varying size obtained through recursive feature elimination. The size of the 'optimal model' varied considerably among different traits. For pubescent leaves, it is evident that the optimal model contains one marker only – indeed the locus determining the character (m*Pub*). All additional markers actually decrease performance as they only add noise rather than information. This effect was also observed for other traits such as yield (not shown). Plant height is an example of a trait that can be accurately modelled with a small number of markers, thus suggesting a relatively low genetic complexity. Diastatic power and *α*-amylase, by contrast, are traits that appear to be genetically quite complex. For example to accurately model diastatic power, 100 markers are required, while 400 markers are required for *α*-amylase. These large numbers suggest that the genetic signal is spread out throughout the genome, and that many markers influence (with small individual effects) the phenotypic outcome.

**Figure 4:**
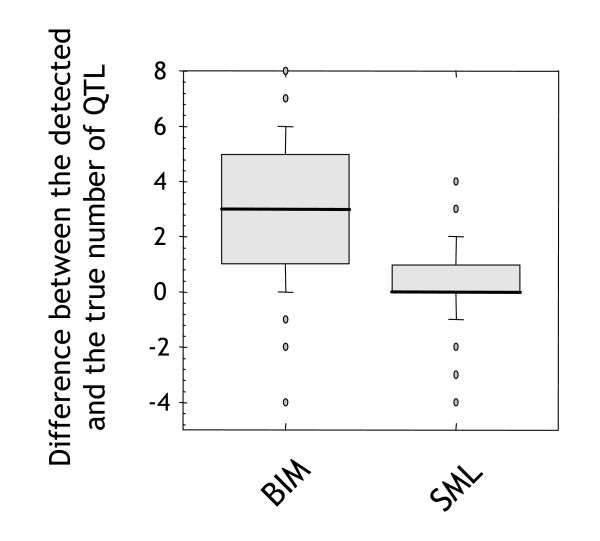
**Accuracy of genetic complexity estimates**. Comparison of an analysis of 100 synthetic datasets with BIM and SML. The y-axis shows the difference between the true and estimated number of QTL.

To verify the accuracy of estimating the number of QTL, we performed simulation experiments using a group of 100 artificial datasets. These datasets were simultaneously analysed by Bayesian Interval Mapping (BIM) [12,13] for the purpose of benchmarking our method. Each dataset contained 1-10 QTL positioned randomly at markers evenly spaced at 1 cM intervals across ten chromosomes of 100 cM length. As shown in Figure 4, the median difference in the number of detected QTL for SML is zero, with a low variance. This result demonstrates that the genetic complexity of traits can be estimated very precisely from the performance curves given by the SML method. By contrast, BIM tends to underestimate the number of QTL.

### Statistical validation of QTL through bootstrapping

An important estimation technique used in our method is bootstrap resampling. Bootstrap resampling involves creating a subset of the data for training, and using the remainder for testing (see *Methods*). In this way, independent data are reserved for testing the model derived from the training data. This approach produces less biased estimates of the generalisation error (the predictive performance of a model on data unseen during training), and hence a better estimate of the true effect of a putative QTL [14].

**Figure 5:**
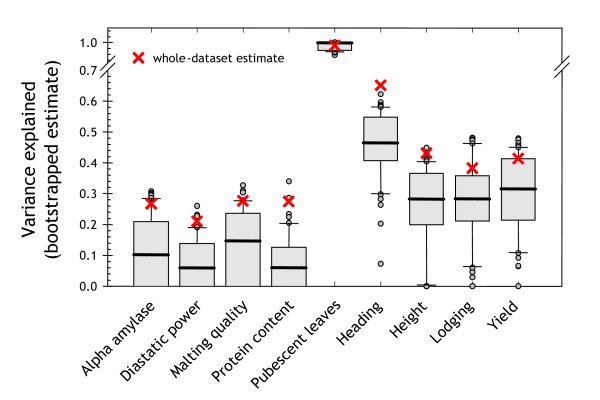
**Whole-dataset bias**. Demonstration of the optimistic bias that arises when measuring predictive performance on training data. For each trait, the optimal marker was selected using MR, either on the entire dataset (red crosses) or within a 200-permutation zero bootstrap environment (box plots).

Figure 5 illustrates the bias that can occur when not using independent DH lines for testing the predictive power of a QTL model. We used MR to detect the top QTL and estimate its predictive performance, both using bootstrap resampling and resubstitution (i.e. deriving an estimate based on the whole dataset). For the bootstrap analysis, 200 iterations were used. Each iteration involved detecting the top QTL using MR and training a single QTL linear model on the training data, then estimating the variance explained on the independent test data (the withheld DH lines). In the figure, the red crosses and box plots show the results obtained with resubstitution and bootstrap resampling, respectively. For each trait except pubescence leaves, the resubstitution estimate is overly optimistic, sitting outside the upper quartile of the bootstrap estimate.

This result illustrates that resubstitution estimates of QTL effects are inherently biased upward. As a consequence, bootstrap resampling reduces the detection of spurious QTL; QTL deemed important on the training set by chance will not reflect the same importance when measured on the test data. Other authors have explored resampling techniques such as cross-validation in the context of QTL detection and evaluation [14], and the biases that arise when not using resampling methods have been well demonstrated. Hence the use of bootstrap resampling in the SML procedure should facilitate more robust QTL detection.

### QTL identified compared to other methods

#### Real data

To further benchmark SML against other QTL mapping methods, we identified QTL for nine traits using SML, single Marker Regression (MR), Composite Interval Mapping (CIM) and BIM. In the case of CIM we used 20 markers at > 10 cM distance from the investigated interval to adjust for the genome background. For BIM, the default values specified in the R/qtlbim package were used for the priors and sampling parameters. Table 2 shows the average degree of correlation of the genome profiles of variance explained (the QTL effects) among the various methods. SML and CIM produced the most correlated results (Pearson's correlation coefficient *r *= 0.80). This is despite the fact that SML uses marker information only, while CIM requires the additional information of a genetic map. The BIM profiles were less correlated with the profiles generated by other methods on average.

**Table 2:** Correlation between genome profiles of variance explained obtained with different QTL-mapping methods.^a^

**Method^b^**	**SML**	**MR**	**CIM**	**BIM**
**MR**	0.65 ± 0.04	-		
**CIM**	0.80 ± 0.09	0.72 ± 0.15	-	
**BIM**	0.48 ± 0.13	0.46 ± 0.15	0.44 ± 0.14	-

^a ^The values given are means ± SD across the nine traits investigated in this study.

^b ^QTL-detection methods were: SML, Statistical Machine Learning; MR, single Marker Regression; CIM, Composite Interval Mapping with 20 background markers at > 10 cM distance from the tested interval, and BIM, Bayesian Interval Mapping.

**Figure 6:**
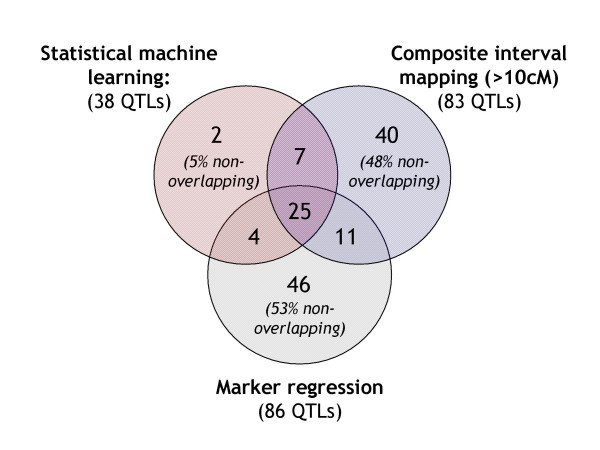
**Cross-validation of QTL**. Overlaps among QTL detected by SML, MR and CIM at a *p *< 0.05 level. QTL in common between each pair of methods were identified as described in the section entitled 'Comparisons between QTL-detection methods and map versions' in *Methods*. The reported numbers are the sums across all nine traits investigated in this study.

We next counted and compared the QTL reported by SML, MR and CIM at a significance level of *p *< 0.05 (Figure 6). BIM was not included in this detailed comparison as it is difficult to match the frequentist null-hypothesis rejection thresholds with the Bayes factors used with BIM. SML reported slightly less than half the number of QTL than MR and CIM, presumably because the bootstrap-validation step eliminated spurious QTL (see previous section); MR, for example, reported five spurious peaks for pubescent leaves, a trait known to be encoded by a single Mendelian trait (Additional File 1). Perhaps not surprisingly, about half of the QTL detected by either MR or CIM could not be cross-validated by a second method. By contrast, 95% of the QTL identified by SML were also detected by MR and/or CIM (Figure 6). These results suggest that QTL detected by SML are more robust and hence more likely to be 'biologically significant'.

There was a large overlap between QTL identified in this study and previous studies of the same DH population [15-18]. SML identified well-known major QTL for *α*-amylase (chromosomes 2 H, 7 H), diastatic power (1 H, 4 H, 7 H), grain protein content (2 H, 4 H, 5 H), malt extract (2 H, 4 H, 7 H), heading date (2 H), height (2 H, 3 H), lodging (2 H, 3 H, 4 H) and yield (3 H) (Additional File 1) [15-17].

**Figure 7:**
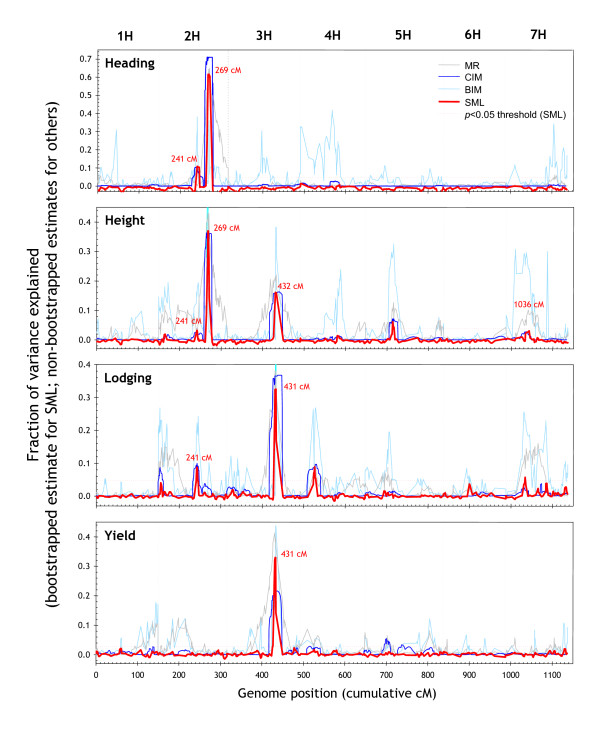
**Comparison of different QTL methods**. Genome-wide QTL profiles for four traits generated by SML, MR, CIM and BIM. A 5 cM averaging window was applied to the BIM profile for plotting. Horizontal dotted lines are *p *< 0.05 thresholds for SML. The plots are based on the allele calls and genotypes underlying the 'raw' version of the linkage map (see section entitled 'Genetic-map construction' in *Methods*).

Figure 7 displays the profiles generated using several methods on the heading date, height, lodging and yield traits. The yield QTL on chromosome 3 H at a cumulative map position of 431 cM indeed coincided closely with the main lodging QTL (431 cM) and one of the plant-height QTL (432 cM). Lodging is expected to affect yield, yet the yield QTL profile produced by SML was identical, irrespective of whether or not environments where lodging was reported were included in the analysis (data not shown).

Hayes and colleagues suggested that the positive allele for the yield QTL on chromosome 3 H coincided with low lodging and height-QTL alleles from the opposite parent [15]. These previous observations are clearly reinforced by our results and appear to point to a locus influencing plant height that has independent pleiotropic effects on both lodging and yield as opposed to a causal chain (tall plants → lodging → reduced yield). Plant height also appeared to affect lodging via another QTL on chromosomes 2 H (241 cM), which coincided for the two traits. Plant height, in turn, appeared to be partly associated with heading date because the main QTL for these two traits coincided precisely (chromosome 2 H; 269 cM). We conclude that the SML-QTL algorithm confirms and extends previously hypothesised relationships among these traits. Clearly, the resolution of the QTL profiles generated by SML facilitates the genetic dissection of traits into physiological or phenological components.

#### Synthetic data

We also compared the genome profiles of variance explained (the QTL effects) derived from the 100 synthetic datasets discussed earlier, in order to benchmark SML against BIM and MR. These methods were selected to represent the two extremes of algorithmic complexity of existing QTL mapping methods. To summarise these profiles and give an idea of overall performance of each method, we considered each dataset to be a binary classification problem – for each marker, classify it as a QTL or not a QTL. Such a binary classification can be accomplished by choosing a threshold and classifying markers exceeding this threshold as linked to QTL. However, as the threshold affects the trade-off between type-I and type-II errors, we used the Area under the Receiver Operating Characteristic (AROC) [19] to measure the performance. The AROC is an order statistic equal to the probability of correctly ordering pairs from different classes (see "QTL classification performance" section in *Methods*).

**Figure 8:**
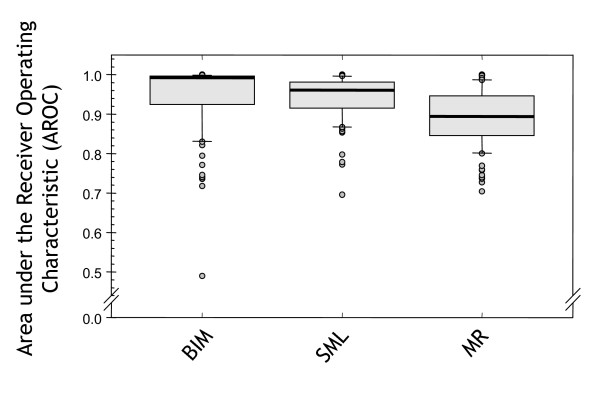
**QTL profile accuracy on simulated data**. Accuracy of different methods of classifying individual markers as linked to synthetic QTL on 100 simulated datasets. Results of genome profiles obtained using BIM, SML, and MR on 100 simulated datasets. The y-axis here is the Area under the Receiver Operating Characteristic (AROC). The 0.5 level indicates random performance and 1 indicates perfect performance.

Figure 8 summarises this experiment in the form of a box plot. The results demonstrate that MR performs worse than BIM and SML – as expected – with a lower median and large variance. BIM achieved a high median performance, but had a larger variance than SML. Though the BIM median was higher, the difference between the means of SML and BIM was not significant (*p *= 0.499). We conclude that both methods are similar with respect to locating QTL.

**Figure 9:**
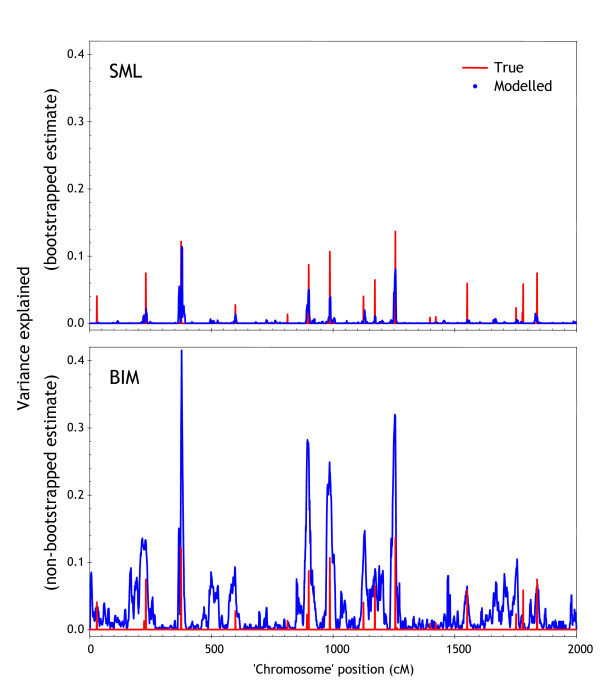
**SML and BIM genome profiles on synthetic data**. Estimated QTL effects using BIM and SML for a single synthetic 'chromosome' of 2,000 cM length with 20 simulated QTL. QTL were positioned randomly with random strength. Red lines indicate true QTL locations, with height denoting strength. BIM profile smoothed using a 5 cM averaging window, and SML profile smoothed using a 5 cM summing window.

Finally, we examined a single synthetic dataset comprising of a 2,000 cM-long 'chromosome' that contained 20 randomly positioned QTL of random strength. Figure 9 shows the smoothed profiles (5 cM averaging window for BIM and 5 cM summing window for SML) of variance explained obtained using BIM and SML (See Additional File 2). Here it is clear that SML provides better estimates of QTL strength – non-QTL markers are assigned low variance explained and the estimates at QTL markers are not overly optimistic. The lack of a bootstrapping step during which experimental units (plants) are resampled presumably accounts for the upward bias of BIM (see also section entitled "Statistical validation of QTL through bootstrapping"). One may claim that SML is underestimating the variance, however after applying the suggested 5 cM summing window the estimates are improved.

It is important to emphasize that the *amount *of variance explained *supportable by the data *will be less than the theoretical variance explained shown in red due to small sample size (100 samples with 2001 features) and noise. Measuring the AROC on both variance explained profiles gives 0.83 for SML and 0.78 for BIM, indicating the SML peaks are better aligned with QTL and more distinct than the BIM peaks.

### QTL resolution

The precision with which a QTL can be mapped is important in the context of marker-assisted selection and gene cloning in particular. Narrow QTL peaks are also important for distinguishing closely linked QTL (or genes) affecting the trait. Figures 7 and 9 demonstrate that SML consistently generated narrower and better defined QTL signals than MR, CIM and BIM. It should be noted that we used quite aggressive settings for CIM to produce narrow QTL peaks (background markers at > 10 cM) [5]. To evaluate the precision of SML, we investigated the centromeric region on chromosome 7 H flanked by markers *Amy2 *(64 cM) and *Brz *(95.2 cM) (Additional File 3). This region contains several overlapping QTL for malting-quality traits, including malt extract, *α*-amylase and diastatic power [15,18].

**Figure 10:**
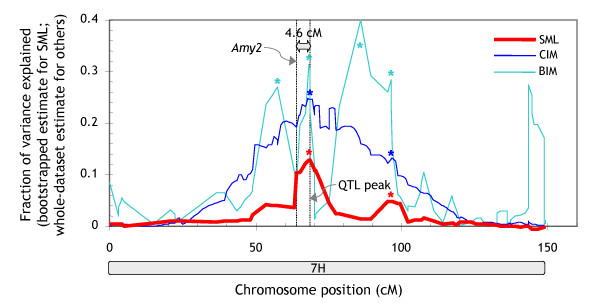
**QTL for *α*-amylase on chromosome 7 H**. QTL profiles produced with SML, CIM and BIM. The positions of the structural α-amylase gene (*Amy2*) and the maximum of the SML QTL peak are indicated by vertical dotted lines. A 5 cM averaging window was applied to the BIM profile for plotting. 'Significant peaks' (*p *< 0.05 for SML and CIM; 2log BF > 2.2 for BIM) are highlighted by asterisks. The plot is based on the allele calls and genotypes underlying the 'raw' version of the linkage map (see section entitled 'Genetic-map construction' in *Methods*).

It had been speculated that one of the two *α*-amylase QTL could be attributed to *Amy2*, a structural gene encoding low-*p*I *α*-amylase [15]. The resolution afforded by conventional QTL-mapping methods, however, was insufficient to settle this issue. The CIM analysis in this study also reported a broad peak on chromosome 7 H. The QTL profile generated by SML, by contrast, showed two distinct peaks (Figure 10; Additional File 1). One of the two peaks was at 4.6-cM distance from the *Amy2 *locus (the other was further away). Given that various partially related traits mapped to identical QTL with less than 1-cM precision (Figure 7), a 4.6-cM distance would suggest the structural gene and the QTL are not identical. This result is indeed consistent with a fine-mapping study of this region that identified recombinants between *Amy2 *and the QTL [18] and hence underscores the high resolution afforded by SML.

Conventional methods map QTL with limited precision, particularly if the fraction of the variance explained by a QTL is low [20]. In CIM, the width of QTL peaks can be reduced by using more closely linked markers for genetic-background adjustment. This approach, however, decreases the statistical power of the method [5] and relies on an *ad-hoc *cM-distance threshold. BIM provides a similar degree of resolution as SML but appears to overestimate QTL effects to an even larger extent than CIM, and reports QTL peaks not supported by the other methods (Figure 10).

By contrast, SML generates unbiased QTL models and increases QTL definition by shrinking the size of the models through recursive marker elimination and apportioning variance to individual markers based on nested models. Individual markers are evaluated in the context of other markers; so if multiple markers contain a similar level of information then the (largely) superfluous markers will be removed. The remaining marker(s) will still explain most of the variance, while the variance attributed to the superfluous markers will be small, thus resulting in well-defined QTL peaks.

### Robustness to genotyping and linkage-mapping errors

Genotyping errors affect the accuracy of the marker order on a genetic map and hence the performance of QTL-detection methods that require a linkage map. We compared the QTL profiles produced with SML, CIM and MR using two different genotypic datasets: the dataset underlying a 'raw' version of the Steptoe/Morex map (0.4% potential genotyping errors; 97.0% call rate) and the dataset corresponding to a 'curated', re-optimised version of the map (potential genotyping errors removed; 99.6% call rate). Table 3 presents an overview of this comparison. The QTL profiles were highly correlated for MR, less correlated for SML and the least correlated for CIM. Despite the high correlated QTL profiles, only 67% of more than 80 QTL identified with MR were consistent between the two map versions. The between-map consistency of the QTL detected with CIM (approximately 80) was even lower (64%).

**Table 3:** Consistency between QTL detected with 'raw' and 'curated' genotypic data.

		**Total number of QTL detected (*p *< 0.05)**		
		
**Method^a^**	**Correlation between QTL profiles^b^**	**Raw dataset**	**Curated dataset**	**Overlap^c^**
SML	0.895 ± 0.085	38	34	29 (81%)
MR	0.998 ± 0.002	86	84	57 (67%)
CIM	0.887 ± 0.128	83	86	57 (67%)

^a ^SML, Statistical Machine Learning; MR, single Marker Regression; CIM, Composite Interval Mapping with 20 background markers at > 10 cM distance from the tested interval.

^b ^QTL profiles are whole-genome plots of the fraction of variance explained vs. genome position similar to those displayed in Figures 6-8. The values reported are means ± SD across the nine traits investigated in this study.

^c^The percentage overlap was computed by division by the average number of QTL detected with the two datasets.

As a result of the bootstrap-validation step, SML reported less than half of the QTL identified by other methods (see section entitled *Statistical validation of QTL through bootstrapping *above). However, 81% of these QTL were consistent between map versions. In contrast to CIM, the SML method can function independently of a genetic map. We only used the map for smoothing and conveniently plotting the results. An erroneous marker order in a linkage map, therefore, affects SML only marginally during the final smoothing/plotting step.

**Figure 11:**
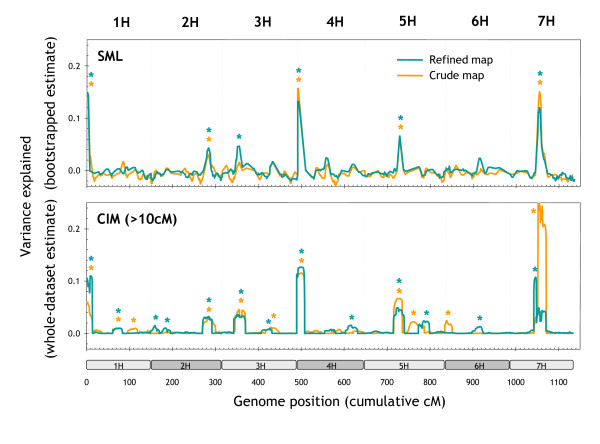
**Robustness to genotyping and linkage-mapping errors**. Effect of map curation on QTL for diastatic power detected by SML and CIM. In the case of CIM, 20 markers at > 10 cM distance from the tested interval were used to adjust for the genetic background. Statistically significant peaks (*p *< 0.05) are labelled with asterisks.

Map curation not only affected QTL detection but also the estimation of QTL effects. Figure 11 displays a between-map comparison for diastatic power, one of the genetically more complex traits. In the case of SML, the variance explained by QTL was consistent between the two datasets. CIM was less consistent. For example, map curation reduced the explanatory power of the most important CIM QTL on chromosome 7H from 25% to 10% of variance explained (Figure 11). We conclude from these results that SML is more robust to genotyping and linkage-mapping errors than both MR and CIM.

Interestingly, the quality of the "crude" genotyping data set used in the analysis reported here is lower than that of a typical dataset produced by a standard DArT assay (see the 'Genotypic data' section in *Methods*) but arguably higher than that of a typical dataset generated with (semi)manually scored markers (AFLP or SSR). From this it follows that:

1. 'Standard' QTL mapping approaches (like CIM), when performed on genotyping datasets obtained with gel-based marker technologies, may produce inconsistent marker/trait associations; and

2. The SML approach is likely to perform well in detecting and estimating QTL effects when using marker data with a quality similar to that of a standard DArT assay, with negligible improvement afforded by either replicating DArT assays or employing technically more complex and costly SNP genotyping platform(s).

## Conclusion

The QTL identified with SML are broadly consistent with those detected by other methods. Yet the SML algorithm offers some advantages over QTL methods such as MR, CIM and BIM. SML produces narrower peaks than MR and CIM and hence identifies QTL with greater precision. BIM generates similarly narrow peaks as SML, but unlike SML seems to underestimate the genetic complexity of traits and overestimate the QTL effects on synthetic data. Because of the use of bootstrap resampling, SML avoids the optimistic bias in predictive performance (% variance explained), which is an inherent feature of other methods. Consequently, SML provides better estimates of the QTL effects supportable by the data, thus reducing the false-discovery rate.

Finally, unlike several other QTL algorithms SML does not require a genetic map. It is therefore applicable to any species or population. Because of this feature, SML is a potentially attractive alternative for association-mapping experiments, an idea that will be explored in a future paper.

## Methods

### Barley population

Our study is based on existing data for 94 F_1_-derived DH plants from a cross between barley cultivars Steptoe and Morex [21-23]. This population has been the subject of extensive phenotyping across a range of environments [22].

### Genotypic data

#### Data source

We used part of the segregation data from a high-quality Steptoe/Morex map with more than 1,000 markers. This map was built from RFLP, DArT and SSR markers [23], and had approximately 0.2% potential genotyping errors. To create a more 'typical' dataset for plant QTL studies reported in the literature (with less markers and a higher error rate), we selected a random subset of 464 markers and added 84 markers with more genotyping errors. The majority of these markers were previously rejected DArT markers with low marker-quality scores [24]. DArT genotypes ('A' for homozygote maternal, 'B' for homozygote paternal) were translated into the original presence/absence allele calls (0/1) by comparison against the parental alleles. RFLP genotypes were converted into presence/absence allele calls by arbitrarily assigning '1' to the maternal allele.

Allele calls (0/1) were used to identify QTL using SML and MR. Missing allele calls were imputed with 0.5 because the ridge regression algorithm underlying our method works on continuous input values (see section entitled QTL *machine-learning algorithm *below). Genotypes (A/B) were used to identify QTL using the map-based CIM approach. Missing genotypes were replaced with expected genotypes derived from flanking markers after genetic-map construction.

#### Genetic map construction

For the purpose of displaying SML results and identifying QTL by CIM, we built a genetic map for the dataset of 548 selected markers (351 DArT, 197 RFLP). The marker order was established with RECORD software, and the cM distances between markers were estimated using a multipoint regression algorithm [25,26]. The resulting 'raw' map had a call rate of 97.0% and contained 0.4% potential genotypic errors (Additional File 3). For comparison, we also generated a 'curated' version of the map. Map curation comprised imputing missing genotypes from neighbouring markers, substituting potential genotyping errors (LOD_error _> 4) [27] with missing data, re-optimising the marker order and collapsing co-segregating markers into 'bins'. The resulting refined map had 367 bins and a call rate of 99.6% (Additional File 3). We used both the 'raw' and the 'curated' allele calls and genotypes to identify QTL.

### Phenotypic data

#### Data source

The phenotypic data for nine traits, measured in up to 16 different environments, were downloaded from the GrainGenes website [22] (Additional File 4).

#### Pre-processing of phenotypic data

We introduce a method strongly related to principal component analysis. Let *p*_*ij *_be the phenotype measurement for plant *i *in environment *j*, *n*_env_, *n*_mrk_, and *n*_*p *_be the number of environments, markers, and plants respectively. Then the mean and standard deviation of phenotypes within environments are given by

$$\begin{array}{l} {\bar{p}}_{j}=n_{p}^{-1}\sum_{i}p_{ij}\\ s_{j}=\sqrt{{\left(n_{p}-1\right)}^{-1}\sum_{i}\left(p_{ij}-{\bar{p}}_{j}\right)} \end{array}$$

where *s*_*j *_and ${\bar{p}}_{j}$ are the sample standard deviation and mean of environment *j *calculated across all plants *i *∈ 1..*n*_*p*_. The scaled phenotypes are then given by

$${\hat{p}}_{ij}=\frac{p_{ij}-{\bar{p}}_{j}}{s_{j}}$$

Finally, we can combine the estimates into a single more robust value by calculating the mean across all environments

$$y_{i}=n_{env}^{-1}\sum_{j}{\hat{p}}_{ij}$$

Note that missing values can be handled during the calculation of *s*_*j *_and ${\bar{p}}_{j}$ by calculating the mean and standard deviation over available measurements only.

These final values *y*_*i *_are very similar to results obtained by projecting onto the first principal component. This can be seen by observing that the *y*_*i *_provide a good linear approximation to the full set *p*_*i,j*_. We verified this on the barley dataset by calculating the principal component projection and measuring the correlation with the values obtained by the above method. The result was a mean correlation coefficient of 0.99 across all traits.

### Synthetic datasets

Synthetic datasets were created using the *R/qtl *package [28]. All datasets were simulated backcrosses using an additive model for the phenotype comprising of 100 individuals. Markers were positioned uniformly across the entire genome with no missing values or genotyping errors. The Haldane mapping function was used to convert genetic distances to recombination fractions. QTL were distributed randomly at marker positions with uniform probability. QTL strength (difference between homozygous and heterozygous) was randomly assigned with uniform probability over the interval [-5,5]. Normally distributed noise with mean 0 and variance 1 was added.

### QTL machine-learning algorithm

The QTL detection algorithm is based on a few key concepts: a linear predictive model, recursive feature elimination, bootstrap resampling for estimation of model performance and marker effects, and generation of QTL profiles by local summation. Figure 1 (left panel) shows a high level overview of the data flow and processing steps involved in generating the QTL profiles. We now detail each concept.

#### Linear predictive model

Underlying our whole technique is the assumption of linear dependence. We assume that contributions from markers are additive. Let *x*_*ij *_be the genotype of plant *i *at marker *j*, and ${\vec{x}}_{i}$ be the vector consisting of all markers from plant *i*. Under the linear assumption, the estimate of *y*_*i *_for plant *i *is

$$f({\vec{x}}_{i};\vec{\beta },b)=\sum_{k\in K}x_{ik}\beta_{k}+b$$

where *K *is a set of markers, *x*_*ik *_is the genotype of marker *k *for plant *i*, $\vec{\beta }$ is the associated weight vector, and *b *is the bias parameter.

The parameters $\vec{\beta }$ and *b *are estimated from the training data using the well-known ridge regression algorithm [29,30]. In brief, ridge regression solves the least squares problem

$$\min\sum_{i}{\left(y_{i}-f({\vec{x}}_{i};\vec{\beta },b)\right)}^{2}+\lambda \sum_{k}\beta_{k}^{2}$$

where the first term is the sum of squares, the second term is the regulariser, and *λ *> 0 is a tuning parameter for adjusting the amount of regularisation. The regulariser encodes a preference for smoother functions by shrinking the weights towards 0 (and also each other), and gives both a unique solution to the ill-posed minimisation problem and increased robustness against noise. For our QTL analyses, we set *λ *= 1.

#### Recursive feature elimination

While a model over the entire set of markers is useful for predicting the phenotypic outcome, we wish to determine the key markers contributing to the genetic variation of traits. In other words, we seek a model with *K *of low cardinality (i.e. with a low number of elements in the set) that is sufficient for accurate phenotype prediction. This feature (marker) selection is performed by using Recursive Feature Elimination (RFE) to train and evaluate linear models ranging in size from all features to one feature.

RFE commences with the full model using all features and then discards the least important feature. This process is recursively applied until a model of desired size is reached (we created models down to one marker). In coupling RFE with ridge regression (RFE-RIDGE), the importance can be estimated from the weights $\vec{\beta }$ = (*β*_*k*_). As the model is linear and all markers have the same range, the absolute value |*β*_*k*_| is an estimate of the importance of the marker *k*. The *k*^th ^marker with minimal |*β*_*k*_| is deemed the least important and is discarded. Note that re-optimisation of $\vec{\beta }$ after each discard is required as the exclusion of a feature will result in a redistribution of weights.

More precisely, let ${\vec{\beta }}^{\left(t\right)}$ be the model obtained at time step *t *from applying ridge regression with the set of markers *M*_*t*_. The initial model at time step *t = 1 *is fitted with all markers *M*_1 _= {1,2,..., *m*}. At each time step, determine the least important feature as $\xi_{t}=arg\underset{k}{\min}\left|\beta_{k}^{\left(t\right)}\right|$. The new set of markers for the next time step is then *M*_*t*+1 _= *M*_*t*_\{*ζ*_*t*_}.

#### Bootstrap resampling

To estimate the performance of models the *ε*-0 bootstrap method was used [31]. As mentioned previously, this method involves sampling the original dataset with replacement to create a training set, and using all remaining un-sampled instances as the independent test set (Figure 1, right panel). The models are then built on the training set, with the test set reserved for the evaluation of model performance. This process was repeated 50 times.

#### Evaluation of models and estimation of marker contributions

To evaluate the performance of a model we used the fraction of variance explained as a criterion. Suppose we have a model ($\vec{\beta }$, *b*) and we wish to evaluate the variance explained on some test set *T*. Then, the variance explained is defined as

$$r^{2}(\vec{\beta },b)=1-\min\left(\frac{\sum_{i\in T}{\left(y_{i}-f({\vec{x}}_{i};\vec{\beta },b)\right)}^{2}}{\sum_{i\in T}{\left(y_{i}-\bar{y}\right)}^{2}},1\right)$$

where $\bar{y}=\frac{1}{\left|T\right|}\sum_{i\in T}y_{i}$. This measure provides an overall estimation of the predictive performance of a given model.

In addition to evaluating the model, a measure of the contribution of individual markers is needed to locate putative QTL. Quantifying these can be done by recasting this problem as a novelty-detection problem: we wish to quantify the amount of additional predictive power provided by each marker given some already selected set of markers. We measure this degree of novelty using the models built with RFE-RIDGE. As RFE-RIDGE produces nested subsets of selected markers, we can attribute the change in variance explained to the marker that was removed between two consecutive models. More precisely, let $m_{l}=({\vec{\beta }}_{l},b_{l})=((\beta_{kl}),b_{l})\in {\text{R}}^{n_{mrk}}\times \text{R}$ be the sequence of models of decreasing size, i.e.{# *j *| *β*_*kl *_= 0} > {# *j *| *β*_*j*(*i*+1) _= 0}, and *d*_*l *_be the marker eliminated between *m*_*l *_and *m*_*l*+1_. Then

Δ*r*^2 ^(*d*_*l*_) = *r*^2 ^(*m*_*l*_) - *r*^2 ^(*m*_*l*+1_)

is a measure of the novelty of a marker with respect to all the remaining markers in the model. We expect that a key QTL marker will be novel in this sense and result in a large change of variance explained when dropped from the model. The average over the bootstrap iterations provides a robust estimate of the importance of each marker to trait prediction. This estimate is referred to as $\bar{\Delta r^{2}(d_{l})}$.

#### Generation of QTL profiles

The information provided by Δ*r*^2 ^(*d*_*l*_) is immediately useful; we can examine which markers are found to have significant contributions. If a linkage map is available, we can use it to create graphs similar to conventional QTL profiles by simply plotting $\bar{\Delta r^{2}(d_{l})}$ vs. the marker positions. However, the $\bar{\Delta r^{2}(d_{l})}$ value of a particular genetic location is sometimes 'spread out' among a few highly correlated (genetically close) markers, due to the linkage disequilibrium between the markers and the QTL. This effect can be reduced by smoothing the results based on the positions of markers on a genetic map; for the experiments on barley we smoothed the curves by applying a summing window of 5 cM to collect the contributions of genetically close markers. The 5 cM size was chosen because it provides a good balance between resolution and smoothness.

Finally, there are two methods for determining a 95% significance threshold. We assume the smoothed $\bar{\Delta r^{2}(d_{l})}$ were gamma distributed. The gamma assumption is justified as previous literature shows that QTL effects are gamma distributed [32], and 95% thresholds can easily be determined by fitting a gamma distribution. Alternatively, when no smoothing is applied an empirical method can be used to estimate the p-values from the bootstrap replicates by applying a standard one-sample t-test.

### QTL classification performance

The Area under the Receiver Operating Characteristic (AROC) [19] is a general measure of classification performance. We used it to evaluate QTL profiles for simulated data where the QTL positions are known. Let *s*_*i *_be a score (for example the apportioned variance explained produced by the SML) for each marker *i, Q *be the set of indices of 'QTL markers' and *N *be the set of indices of 'non-QTL markers.' The AROC is then given by

$$P(s_{i}>s_{j}|i\in Q,j\in N)+\frac{1}{2}P(s_{i}=s_{j}|i\in Q,j\in N)$$

Given a finite set of scores the AROC can simply be estimated by counting:

$$\frac{1}{\left|Q\right|\left|N\right|}\sum_{i\in Q,j\in N}\{\begin{array}{cc} 1 & \text{if }s_{i}>s_{j}\\ 0.5 & \text{if }s_{i}=s_{j}\\ 0 & \text{otherwise} \end{array}$$

### Other QTL-mapping methods

#### Single Marker Regression (MR)

To obtain the fraction of variance explained for individual markers, the Pearson correlation coefficient between the marker and the phenotype was squared. A phenotype permutation test of 1,000 iterations was used to derive empirical 95% significance thresholds for genome profiles of variance explained [33].

#### Composite Interval Mapping (CIM)

QTL were also identified by CIM using Cartographer 2.5 software [5,35,36]. The program settings were adjusted to scan the genome at a walk speed of 1 cM. The 20 most important markers, selected by forward stepwise regression outside a 10-cM window on either side of the markers flanking the test site were used to adjust for the genetic background [36]. Experiment-wise 95% significance threshold for likelihood-ratio genome profiles were estimated using a permutation test based on shuffling genotypes against phenotypes [33,37].

#### Bayesian Interval Mapping (BIM)

Finally, SML was also benchmarked against BIM [12] using the R package *qtlbim *[13]. The algorithm was restricted to analysis at marker positions only and not within intervals. Two types of genome profiles were used in experiments – Bayes Factor (BF) profiles for QTL detection, and 'heritability profiles' (i.e. variance explained) for estimating QTL effects. The number of QTL was also estimated using Bayes factors.

### Comparisons of QTL profiles

The QTL profiles generated by different methods were compared by computing the Pearson correlation coefficient between the genome profiles of variance explained. For the comparison between different map versions (comprising unequal numbers of markers or bins), the genome scans were first approximated by loess curves based on 1,000 evenly spaced loci.

Statistically significant QTL were identified for each method by recording the cM positions of peak maxima in genome-wide plots of variance explained (*p *< 0.05). Each contiguous stretch of above-threshold markers was considered to belong to a single QTL peak. Small clusters of above-threshold markers at less than 5 cM distance from such a stretch of markers (if present) were considered to be part of the shoulder of the same QTL peak. The overlap between the sets of QTL identified using different methods (or map versions) was quantified by counting the instances in which they detected significant QTL within 10-cM of each other.

## List of abbreviations

BF, Bayes Factor; BIM, Bayesian Interval Mapping; CIM, composite interval mapping; DArT, diversity arrays technology; DH, doubled haploid; LOD score, logarithm-of-odds ratio in favour of a QTL; LOD_error_, logarithm of odds value in favour of genotyping error; MIM, multiple interval mapping; MR, single marker regression; QTL, quantitative trait locus/loci; RFE, recursive feature elimination; RFE-RIDGE recursive feature elimination – ridge regression; RFLP, restriction fragment length polymorphism; SIM, simple interval mapping; SML, statistical machine-learning; SSR, simple sequence repeat.

## Authors' contributions

JB developed and tested the SML algorithm and phenotype pre-processing procedure, performed the BIM analyses and drafted part of the manuscript. PW provided intellectual input during the development and testing of SML algorithms, built the Steptoe/Morex map, performed the CIM analysis, compared the results of the various QTL methods and drafted part of the manuscript. AKo supervised the development of the SML algorithm and co-edited the manuscript. AKi provided intellectual input during the development and testing of SML algorithms and designed and drafted part of the manuscript. All authors read and approved the final manuscript.

## Supplementary Material

Additional file 1 **QTL detected with different algorithms (*p *< 0.05)**. PDF file containing a list of QTL identified for each combination of QTL-detection method (SML, MR, and CIM) and trait (*α*-amylase, diastatic power, heading date, plant height, lodging, malt extract, pubescent leaves, grain protein content, and yield). Click here for file

Additional file 2 **Unsmoothed results obtained in the analysis of a synthetic 'chromosome'**. PowerPoint file with two plots containing the unsmoothed results from which the plots in Figure 9 were generated. Click here for file

Additional file 3 **Genotypic data used for QTL analysis**. Excel file containing 0/1 allele calls and A/B genotypes (segregation data) for both the 'raw' and the 'curated' Steptoe/Morex genetic map. Click here for file

Additional file 4 **Phenotypic data used for QTL analysis**. Excel file containing phenotypic data for the nine traits investigated in this study (*α*-amylase, diastatic power, heading date, plant height, lodging, malt extract, pubescent leaves, grain protein content, and yield). The data is from up to 16 different environments and includes averages across standardised environments (see section entitled 'Pre-processing of phenotypic data' in *Methods*). Click here for file
