# Supplementary material for: Precision-mapping and statistical validation of quantitative trait loci by machine learning
Source: BMC Genet. 2008 May 2;9:35. doi: 10.1186/1471-2156-9-35 (PMC2409372; doi:10.1186/1471-2156-9-35)
Supplement: Additional file 1 — QTL detected with different algorithms (p < 0.05). PDF file containing a list of QTL identified for each combination of QTL-detection method (SML, MR, and CIM) and trait (α-amylase, diastatic power, heading date, plant height, lodging, malt extract, pubescent leaves, grain protein content, and yield). [file 1471-2156-9-35-S1.pdf]

Additional File 1. List of QTL identified using *Statistical Machine Learning* (SML), single *Marker Regression* (MR) and *Composite Interval Mapping* (CIM). The analyses were performed using the segregation data of the crude map (Additional File 2).

Trait	SML	MR	CIM >10cM
a-Amylase	*1H (113.8), 2H (93.1), 3H (20.6), 5H (68.2, 94.4, 183.9), 7H (68.6, 96.4)*	*1H (96.3, 113.8), 2H (84.9), 3H (36.2), 4H (127.1), 5H (68.1, 135.4, 178.0), 6H (23.7), 7H (68.6, 137.3)*	*1H (126.4), 2H (84.9), 3H (8.9, 25.0, 115.3), 4H (127.1), 5H (65.7, 94.4, 182.8), 7H (68.6, 96.5)*
Diastatic power	*1H (1.5), 2H (134.4), 3H (38.5), 4H (1.1), 5H (80.8), 7H (67.0)*	*1H (1.5, 57.6, 108.1), 2H (83.7, 131.0), 3H (36.2, 78.9), 4H (1.1, 127.1), 5H (68.5), 6H (36.5, 77.2), 7H (67.0)*	*1H (1.8, 71.7), 2H (10.4, 35.2, 135.4), 3H (36.2, 112.1), 4H (1.1, 127.1), 5H (79.8, 140.4), 6H (80.4), 7H (61.5)*
Malt extract	*2H (133.2), 4H (86.6), 5H (179.1), 7H (68.6)*	*1H (1.5, 48.9), 2H (78.6, 93.1, 134.4), 3H (168.9), 4H (90.1), 5H (127.2, 177.2), 6H (23.7), 7H (68.6)*	*1H (1.7, 50.1, 132.0), 2H (4.2, 128.9), 3H (22.8), 4H (90.6, 145.7), 5H (99.3, 181.6), 7H (23.8, 70.1)*
Heading date	*2H (91.2, 117.8)*	*1H (1.1), 2H (71.9, 121.3), 7H (120.0)*	*1H (135.9), 2H (91.2, 121.3), 3H (86.6, 173.3), 4H (0.0, 85.5), 7H (107.7)*
Plant height	*2H (117.8), 3H (117.4), 5H (68.1)*	*1H (35.5, 91.1), 2H (14.8, 46.7, 118.9), 3H (117.4), 4H (142.2), 5H (71.5, 177.2), 6H (149.4), 7H (53.4)*	*2H (22.5, 91.2, 120.7), 3H (117.4), 4H (78.6, 92.6), 5H (68.1), 6H (144.1), 7H (52.8)*
Lodging	*2H (5.9, 95.1), 3H (115.3), 4H (34.1), 6H (60.6), 7H (46.6)*	*2H (15.5, 94.1), 3H (115.3), 4H (35.7, 51.7, 60.5, 142.2), 5H (48.3), 7H (77.6)*	*2H (5.2, 94.2, 112.5), 3H (16.3, 42.1, 117.4), 4H (39.6), 5H (13.0, 71.5), 6H (97.3), 7H (46.5, 89.0, 143.4)*
Pubescent leaves	*3H (38.5)*	*2H (82.7, 143.7), 3H (38.5), 4H (127.1), 5H (0, 10.8, 131.4)*	*3H (42.1)*
Grain protein content	*2H (3.8, 93.1), 3H (117.4, 158.4), 4H (34.1), 5H (46.5, 137.1)*	*2H (95.1), 3H (74.5, 115.3), 5H (37.6, 51.3, 125.6, 133.0), 6H (104.9)*	*2H (46.7, 94.2), 3H (78.9, 138.9, 160.1), 4H (41.6, 58.7), 5H (51.3, 84.2, 136.5), 7H (49.8, 118.7)*
Yield	*3H (115.3)*	*1H (113.8, 135.9), 2H (66.3), 3H (115.3), 4H (18.6, 42.7), 5H (1.1, 54.4, 156.8), 6H (9.8), 7H (22.5, 117.6)*	*3H (117.4), 4H (42.7), 5H (54.5, 93.6)*
