# Supplementary material for: Precision-mapping and statistical validation of quantitative trait loci by machine learning
Source: BMC Genet. 2008 May 2;9:35. doi: 10.1186/1471-2156-9-35 (PMC2409372; doi:10.1186/1471-2156-9-35)
Supplement: Additional file 2 — Unsmoothed results obtained in the analysis of a synthetic 'chromosome'. PowerPoint file with two plots containing the unsmoothed results from which the plots in Figure 9 were generated. [file 1471-2156-9-35-S2.ppt]

## Slide 1
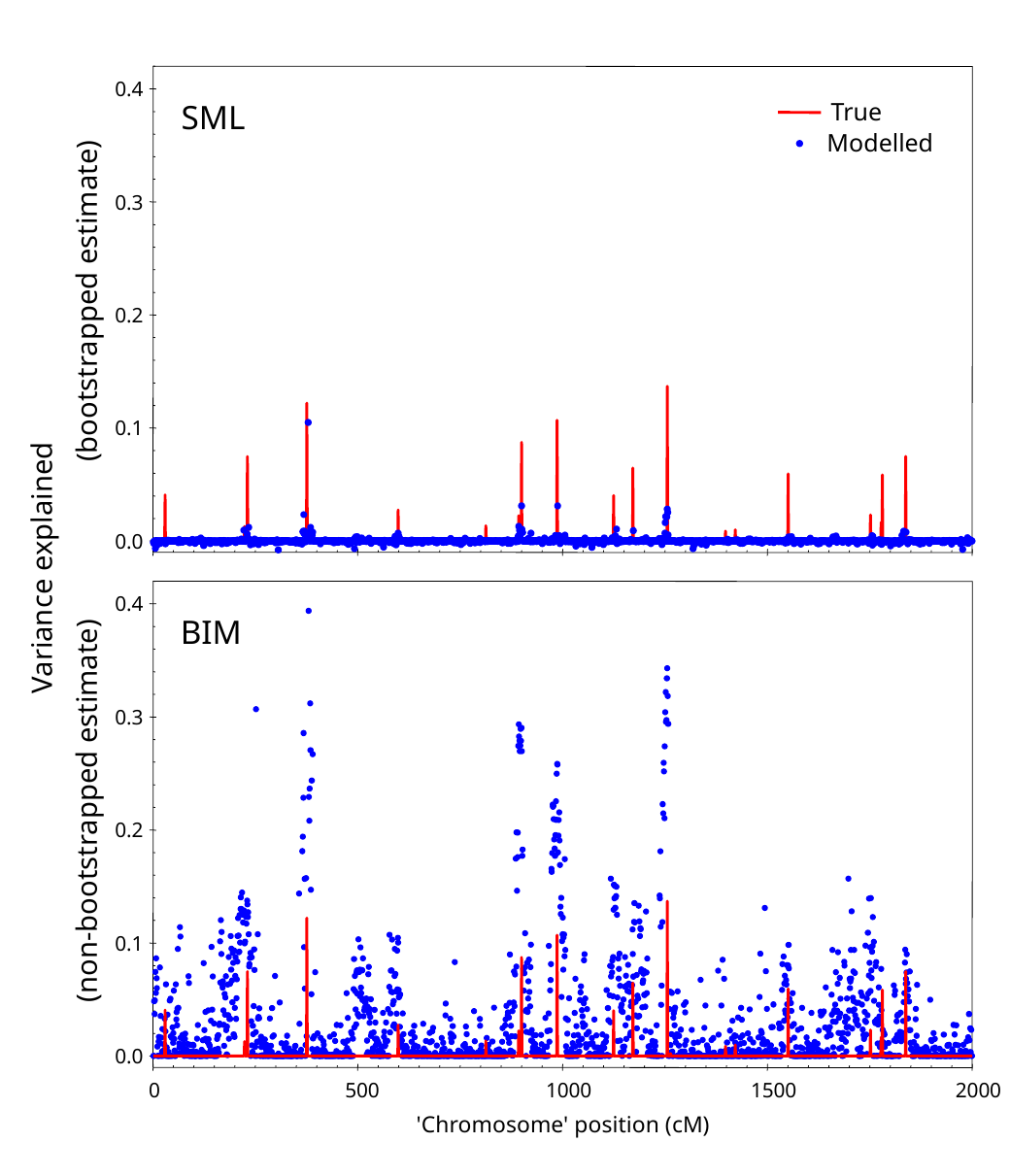

0.4
True
SML
Modelled
0.3
(bootstrapped estimate)
0.2
0.1
0.0
Variance explained
0.4
BIM
0.3
(non-bootstrapped estimate)
0.2
0.1
0.0
0
500
1000
1500
2000
'Chromosome' position (cM)
